# Research on Best Solution for Improving Indoor Air Quality and Reducing Energy Consumption in a High-Risk Radon Dwelling from Romania

**DOI:** 10.3390/ijerph182312482

**Published:** 2021-11-26

**Authors:** Ion-Costinel Mareș, Tiberiu Catalina, Marian-Andrei Istrate, Alexandra Cucoș, Tiberius Dicu, Betty Denissa Burghele, Kinga Hening, Lelia Letitia Popescu, Razvan Stefan Popescu

**Affiliations:** 1 Faculty of Building Services, Technical University of Civil Engineering, 66 Pache Protopopescu Blvd., RO-021414 Bucharest, Romania; andrei.istrate85@gmail.com (M.-A.I.); lelialetitia@yahoo.com (L.L.P.); razvan22@yahoo.com (R.S.P.); 2 Faculty of Environmental Science and Engineering, Babes-Bolyai University, 30 Fântânele Street, RO-400294 Cluj-Napoca, Romania; dinualexandra2007@gmail.com (A.C.); tiby_dicu@yahoo.com (T.D.); burghele.bety@ubbcluj.ro (B.D.B.); szacsvaikinga@gmail.com (K.H.)

**Keywords:** energy consumption, indoor air quality, radon mitigation, simulation program, experimental measurements

## Abstract

The purpose of this article is the assessment of energy efficiency and indoor air quality for a single-family house located in Cluj-Napoca County, Romania. The studied house is meant to be an energy-efficient building with thermal insulation, low U-value windows, and a high efficiency boiler. Increasing the energy efficiency of the house leads to lower indoor air quality, due to lack of natural ventilation. As the experimental campaign regarding indoor air quality revealed, there is a need to find a balance between energy consumption and the quality of the indoor air. To achieve superior indoor air quality, the proposed mitigation systems (decentralized mechanical ventilation with heat recovery combined with a minimally invasive active sub-slab depressurization) have been installed to reduce the high radon level in the dwelling, achieving an energy reduction loss of up to 86%, compared to the traditional natural ventilation of the house. The sub-slab depressurization system was installed in the room with the highest radon level, while the local ventilation system with heat recovery has been installed in the exterior walls of the house. The results have shown significant improvement in the level of radon decreasing the average concentration from 425 to 70 Bq/m^3^, respectively the carbon dioxide average of the measurements being around 760 ppm. The thermal comfort improves significantly also, by stabilizing the indoor temperature at 21 °C, without any important fluctuations. The installation of this system has led to higher indoor air quality, with low energy costs and significant energy savings compared to conventional ventilation (by opening windows).

## 1. Introduction

Air distribution can have a significant impact on different indoor environmental factors, like indoor air quality, energy efficiency, or thermal comfort. Thus, a careful correlation of those parameters at the interior of a building would be needed for a sustainable future. The energy used in the building sector represents around 20–40% of the energy used worldwide, hence a major need to lower these values appeared through the introduction of the energy certification or building to DIRECTIVE (EU) 2018/2002 that set to diminish those values with 32.5% by 2030 [1]. Likewise, 36% and 38% of the CO_2_ emissions are associated with the energy consumption in buildings in Europe [2].

Multiple factors can influence the energy consumption of a building including the size, construction materials, orientation, shape factor, or air infiltrations [3]. The study showed that almost 65% of the total primary energy consumed by a building situated in China is used by the heating system [4]. Furthermore, according to Ruano [5], the orientation and the geometrical characteristics of a building could lead to a reduction of energy consumption up to 40% [6]. The meteorological parameters had also shown a great impact on energy consumption through factors like direct radiation, temperature, wind speed, or relative humidity [7]. Moreover, from the geometrical characteristics point of view, it has been proven that tall buildings are more energy-consuming than the smaller ones presenting the same floor area [8]. Furthermore, the glazing type or the ratio between windows and walls represent the most important parameters that can lead to an impact on energy consumption [9]. On the other hand, air infiltration and occupant’s behavior can also have a powerful impact on energy consumption leading up to 50% of the heat losses of a building [10,11]. Occupants’ behavior also represents a parameter that affects the energy consumption of the building. According to Yan [11].

Whilst being preoccupied with lowering the energy consumption and achieving a high energy efficiency building, the proportion of energy used for ventilation in comparison with the total energy consumption is expected to increase as well [12]. The expected low air permeability of a building to reduce heat losses can have an important impact on Indoor Air Quality (IAQ) [13]. Poor ventilation inside a building most commonly leads to high concentrations of carbon dioxide (CO_2_), which represents one of the key factors when discussing indoor air quality [13]. According to Romanian Standard for ventilation, it is recommended that the indoor environment level of CO_2_ be around 800–1000 ppm. [14]. Furthermore, other parameters such as relative humidity, volatile organic compounds, radon concentration, and thermal comfort can play a significant impact in indoor resting environments also [15]. Studies revealed that during the winter period, indoor radon concentrations could grow by a factor of around two for Romania due to the need for energy conservation and lower ventilation rates [16]. Studies showed that sleeping environments are usually described from low change rates leading to a high concentration of pollutants like PM_2.5_ and VOCs above the standardized values [17]. As people spend around 60–90% of their life in enclosed spaces [18], respecting the standards and norms becomes a more and more concerning issue to be taken into consideration. Furthermore, the impact of IAQ on people’s life was highlighted by the World Health Organization (WHO) which showed that around 99,000 deaths in Europe were attributed due to indoor air pollution only in 2012 [19]. Lung cancer was established as being the most common cancer type worldwide presenting approximately 1.6 million deaths annually and radon exposure was linked to it, being the second cause of lung cancer after smoking [20].

Radon is a natural radiative gas produced by radioactive decay of radium–226 [21]. Radon does not react with other elements or form other chemical compounds since it is a noble gas [22]. Once it reached the outdoor atmosphere it is diluted by the other gases and presents lower concentrations. On the other hand, after penetrating the building’s envelope, the concentration can grow to dangerous levels until the ventilation system will dissipate it. Hence, the indoor radon concentrations vary with the internal surfaces and volumes of the enclosed spaces and the air change rate [23].

The preferred method for newly built houses is the installation of an anti-radon membrane along the entire area of the building. In areas with high concentrations of radon in the soil, this method can be supplemented with natural ventilation of the space under the floor, in case of suspended floors, or by installing an active depressurization system [24,25].

The specific method to prevent the high values of radon used in Norway, correlated with the installation of ventilation systems in almost all new buildings, has led to a significant reduction in radon concentrations. This improvement was best observed for individual dwellings [26].

Ensuring proper ventilation into an indoor space, leads to a significant improvement in the indoor air quality, removing some pollutants from the indoor air [27]. For instance, during radon concentration measurements on schools, some differences were found between the active and passive measurements, most probably based on the ventilation strategies and the human behavior [28].

The efficiency of the heat recovery ventilation systems has been demonstrated in several studies, generating significant energy reduction compared to the traditional ventilation method (windows opening) [29].

Although numerous studies have shown that the ventilation of the space ensures good quality of the indoor environment and can even decrease the level of radon in homes. This method can be improved by other techniques, such as the installation of active floor depressurization systems. Studies have shown a good efficiency of these systems [30,31].

The indoor high radon concentration consequences on occupant health have been highly debated to establish optimal mitigation solutions. According to developed research, some types of soils may have the most important contribution to the radon concentration level inside the dwellings [32]. The first mitigation method considered for the pilot dwelling was the Sub-slab depressurization system. To install the equipment composing it, the floor was removed, giving the possibility to install a radon-proof membrane. Studies have shown an efficiency of this method when the limit concentration level is not very much exceeded, or the house is in construction and the membrane can be installed properly [33]. Moreover, many studies have shown the effectiveness of radon-proof membrane-like secondary radon protection [34]. The most used mitigation method, used especially when high radon concentration was measured, is the active/passive sub-slab depressurization method, showing high radon mitigation efficiency [35,36].

The negative impact of energetic refurbishment intervention, which may affect the indoor air quality, respectively the indoor radon concentration [37], could be resolved using another optimal radon mitigation solution by installing a ventilation system, which is very effective when the limit radon value is not very much exceeded [38,39]. To improve the radon mitigation results, these methods were combined, optimizing the results.

In this article, we mitigate the high radon level using two energy-efficient systems. The novelty of this study is represented by the combined mitigation method used through energy-efficient equipment, removing other pollutants from indoor air. The indoor comfort is improved.

This method presents some advantages related to indoor air quality improvement by removing many pollutants from the dwellings, not only mitigating the radon. If the ventilation equipment has a heat exchanger to recover the energy in the ventilated air is even better, achieving four results: radon mitigation, good indoor air quality by removing the pollutants, good interior comfort by keeping constant the indoor temperature and finally, good energy efficiency by recovering the heat from the exhausted air and transferring it to the intake air. All these represent the subject of the following research, combining two radon mitigation methods and analyzing indoor comfort, indoor air quality, radon mitigation, and energy efficiency.

## 2. Pilot Building Description

**Figure 1:**
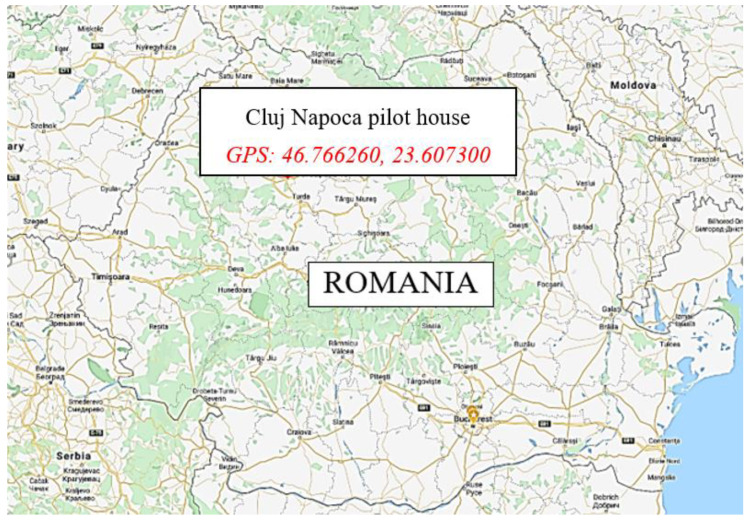
Location of the pilot building.

The studied building is placed in Cluj-Napoca city, ROMANIA (see Figure 1) and presents a ground floor and an attic, forming together a total area of 275.95 m^2^, while the heated area is 239.96 m^2^. The construction was built in 1937, but during this time it had suffered massive changes, including a deep thermal renovation. The brick walls were insulated with a 10 cm layer of expanded polystyrene and the old frame wood double-pane windows were replaced with double glazing PVC windows presenting high thermal resistance of 0.77 m^2^K/W and low air infiltrations. The house is heated using a natural gas boiler connected to the steel panel radiators, installed into the rooms.

The construction presents multiple rooms, but it is mainly occupied by 3 persons (2 adults and 3 children). The lighting system is composed of fluorescent and incandescent sources comprising an installed power of around 1200 W. The geometrical characteristics of the house were grouped in Table 1. It was determined the surface for every construction material (exterior walls, roof surface, windows and doors, floors, etc.) Based on the interior dimensions of the construction, it was determined the heated area and likewise, the heated volume.

**Figure 2:**
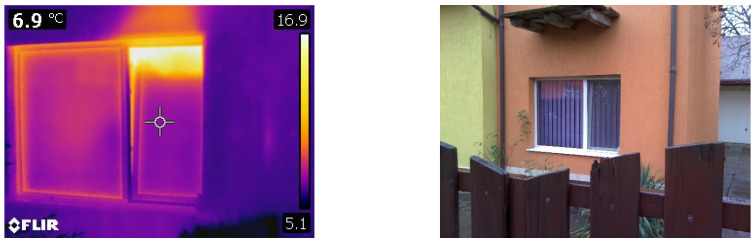
Heat losses from natural ventilation shown by thermography images.

To reduce the indoor radon concentration, the house occupants opened the windows. To highlight the unnecessary heat losses through natural ventilation, a series of determinations were made by thermography. These can be observed quite clearly in Figure 2.

**Figure 3:**
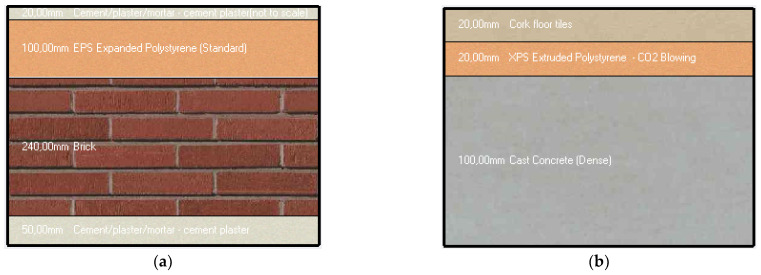
Section in the exterior walls (**a**) and internal floor (**b**) of the presented house.

The structure of the exterior walls is presented in the image below (Figure 3), having the following characteristics:

- Thickness (m): 0.41 m
- U-value surface to surface (W/m^2^-k): 0.341
- R-Value (m^2^-k/W): 3101

The characteristics of the internal floor are:

- Thickness (m): 0.14
- U-value surface to surface (W/m^2^-k): 1.034
- R-Value (m^2^-k/W): 1237

The exterior windows are made by double glazing PVC frame and dividers, has the following characteristics:

- Total solar transmission (SHGC): 0.691;
- Direct solar transmission: 0.624;
- Light transmission: 0.744;
- U–value: 2.6 (W/m^2^-k).

## 3. Numerical Evaluation of Energy Consumption

**Figure 4:**
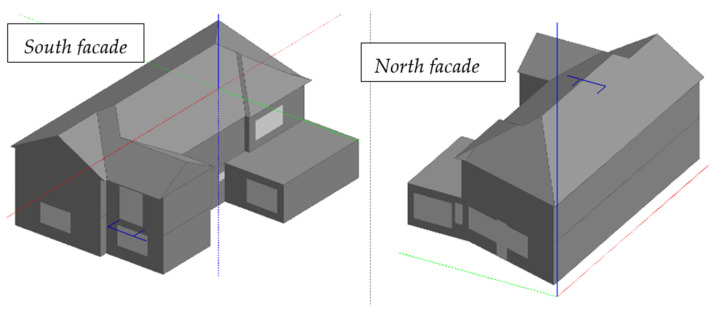
Modeling the pilot building in 3D.

This part of the project was conducted using Design-Builder Version 6.1.7.007 software and Energy Plus 9.01 [40] which can provide the possibility of analyzing the building from the dynamic point of view. The first step was the elaboration of the analyzed construction inside the software based on the real design of the house. Furthermore, the construction materials of the house were set in accordance with the real situation (see Figure 4).

The second step was represented by defining the thermal zones for each floor. On the ground floor, there were set zones regarding two bedrooms (temperature set was 21 °C), a bathroom, a garage, and the living room, whilst for the first floor, it was elaborated one bathroom and other two bedrooms.

**Figure 5:**
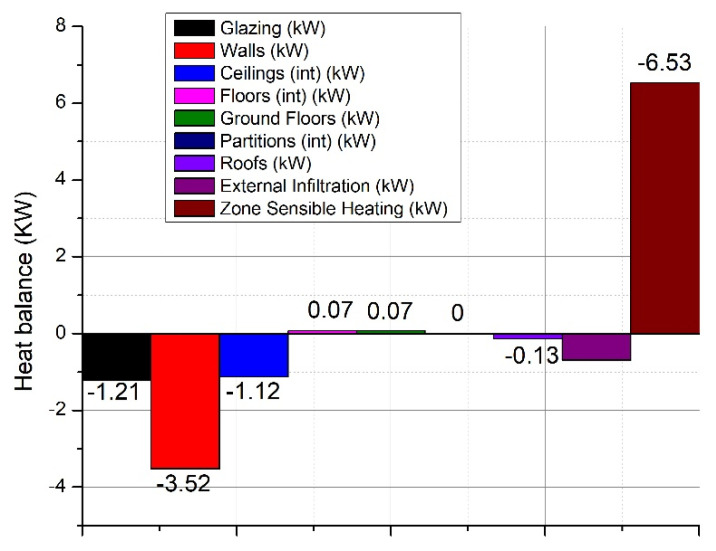
Internal/external heat losses of the studied building.

Furthermore, the meteorological data including the exterior temperature, wind speed, wind direction, exterior pressure, or solar radiation were set for the region where the construction was found for a whole simulation year (Latitude–46.78°, Longitude–23.570°, ASHRAE climate zone 5A). Having all the data set, the next step was to extract the results from the simulation program. The maximum heating load of the construction presents values of approx. 6.52 kW (see Figure 5 for details).

The infiltrations have also significant importance on the heating loads being necessary around 0.69 kW for heating the cold air entering the building (for 0.1 air changes/hour). Moreover, a large amount of heat loss is due to the walls with values around 3.52 kW and through glazing surfaces with values of 1.21 kW.

Moreover, simulations were also performed in case of natural ventilation of the house, by opening the windows, to establish heat losses through the natural ventilated air. To ensure the natural ventilation of the house the windows were opened twice a day, in the morning and the evening. For simulation, an average value of 4 air changes per hour was chosen [41].

**Figure 6:**
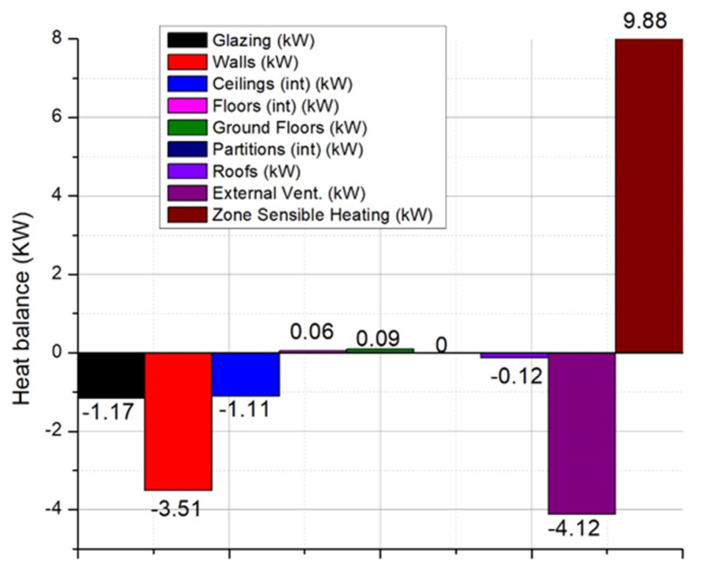
Internal/external heat losses of the studied building with natural ventilation.

Figure 6 shows more clearly the significant impact that natural ventilation, without heat recovery, has on the thermal load of the house.

As we can see from Figure 5, the energy losses through the ventilated air represent about 83% of the total thermal load of the house. To optimize and limit these heat losses, a series of energy-efficient equipment has been researched to improve the quality of the indoor environment, an aspect that will be presented in the following chapters. Another simulation was performed considering the mechanical ventilation of the space with heat recovery ventilation equipment. A rate of 0.6 air exchanges per hour was considered, enough to ensure the minimum of fresh air for the occupants of the home. The heat recovery efficiency of the ventilation equipment was 86% (average known efficiency).

**Figure 7:**
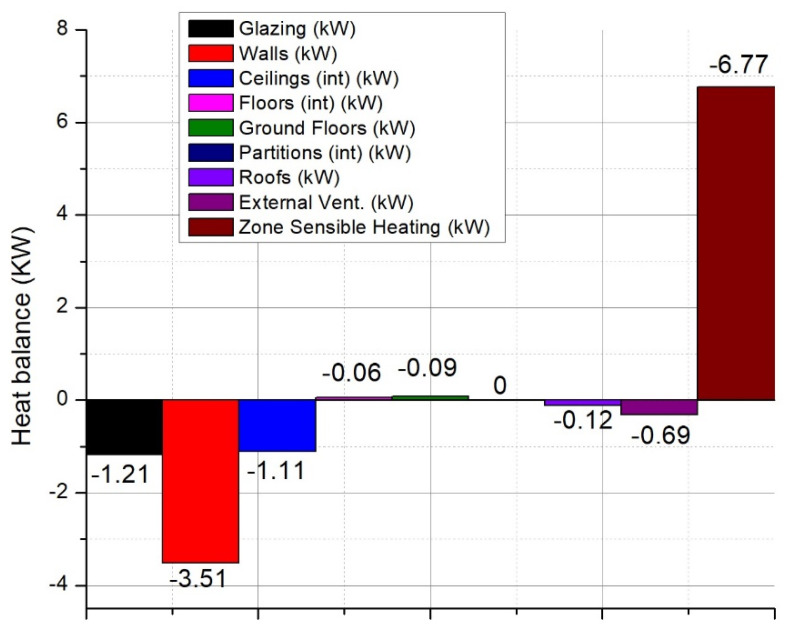
Internal/external heat losses of the studied building with a heat recovery ventilation system.

As it can be seen in Figure 7, the use of ventilation equipment with heat recovery does not significantly affect the thermal load of the house and limits the unnecessary energy losses due to ventilation by opening windows. Improved indoor air quality is maintained continuously, with no fluctuations in indoor pollutant levels or indoor temperature. Ensuring the quality of the indoor environment through mechanical ventilation with heat recovery is the most efficient method. The functioning schedule used for numerical simulation was:

- Weekdays SummerDesignDay, until: 09:00, full flow, until: 20:00, half flow, until: 24:00, full flow;
- WinterDesignDay, until: 09:00, full flow, until: 20:00, half flow, until: 24:00, full flow.

## 4. Experimental Evaluation of Indoor Environment and Energy Consumption

**Figure 8:**
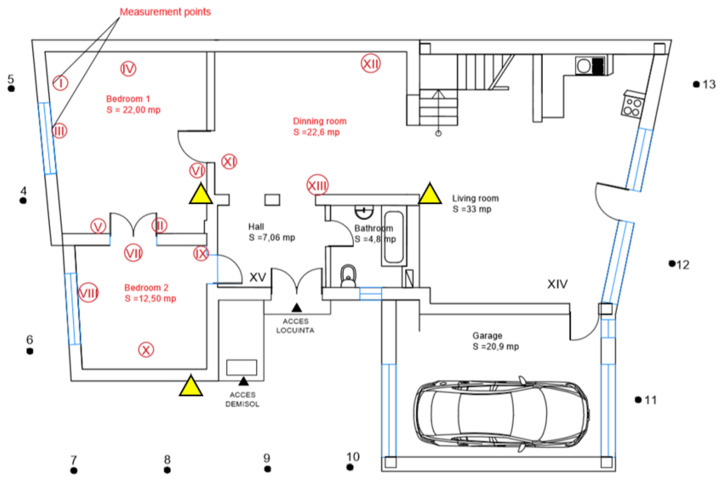
ICA System placed in three different rooms located on the ground floor.

For this session, the indoor air quality for multiple rooms within the pilot house was assessed using the prototype device, namely ICA System [42]. Therefore, several indoor air parameters were measured in three different rooms located on the ground floor, namely the living room and the two bedrooms, bedroom 1 is occupied by the parents, and bedroom 2 is occupied by one child [43]. The analyzed rooms were highlighted with a yellow triangle that can be seen in Figure 8. The main sensor is a high-performance radon sensor capable to measure radon concentration once every 4 min and performing short and long-time moving averaging. The additional sensor also integrated within the ICA System includes sensors for CO, CO_2_, VOC gases and atmospheric pressure, temperature, and relative humidity. Details and technical specifications are found below in Table 2.

The ICA system is an Internet of Things device permanently communicating with a dedicated server. Users can control their ICA System through their phone using a dedicated application. Push notifications or SMS alerts are also available for the users if certain pre-set values on one of the sensors are reached. The ICA system is also able to control a ventilation system to improve the indoor air quality inside the house.

The measurement campaign took place for an entire winter period, but to better express the parameters, a short period was presented, more precisely between 22 January 2019 until 29 January 2019, for a better determination of the indoor air pollutants.

**Figure 9:**
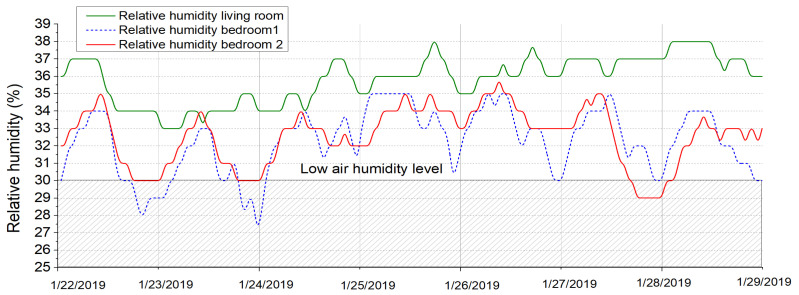
Relative humidity measurements.

**Figure 10:**
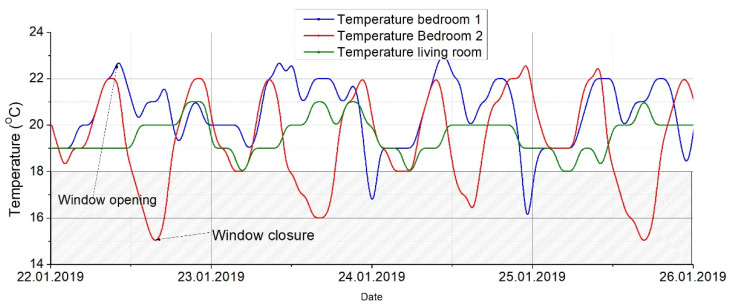
Air temperature measurements.

Figure 9 and Figure 10 show the evolution of the indoor air temperature and the relative humidity during the measurement campaign for all three rooms analyzed. It can be seen the fact that the indoor air temperature presents values evolving from 15 °C up to 23 °C for different periods of time with a mean value around 21 °C, which is the set point value in the two bedrooms, respectively 20 °C in the living room. The moments when the windows were open the air change rate raised and therefore, we had a sudden drop in the indoor temperatures. The graphic also reveals the fact that the parents’ bedroom has a higher rate of windows openings than the other two rooms. Nonetheless, the second graphic is showing the evolution of the relative humidity which presents almost constant values for the rooms around 30% which represents dry air that can cause multiple respiratory problems. Normally the air humidity should be in the range of 40–60% for best comfort. On the other hand, it could be highlighted that the living room has higher values of relative humidity. This could be since the kitchen is communicating with the lounge and the kitchen usually has a higher level of moisture released from the food and its preparation stages.

**Figure 11:**
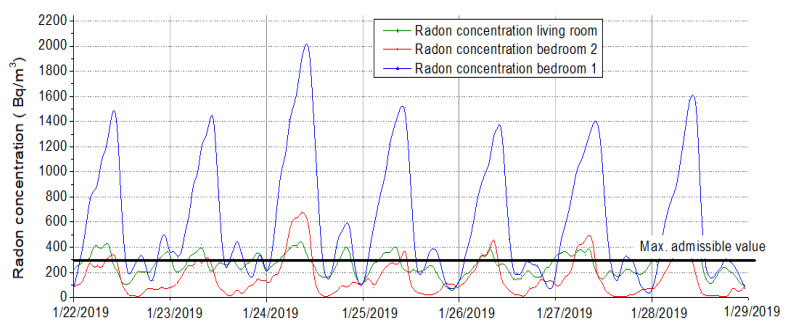
Diurnal variation of radon activity concentration for the three monitored rooms.

**Figure 12:**
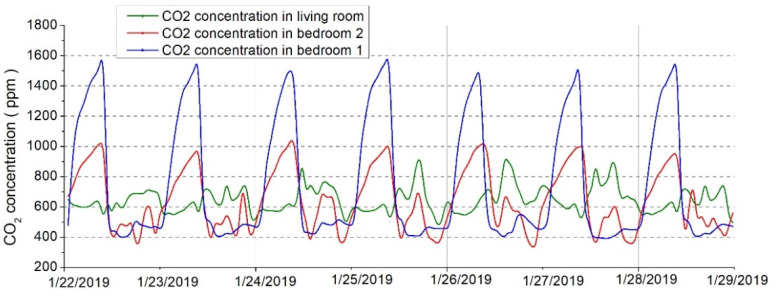
Carbon dioxide levels variations inside the analyzed pilot dwelling.

Figure 11 shows the indoor air concentrations of radon during the experimental campaign for the three rooms analyzed [44]. It can be observed that the child’s bedroom (bedroom 2) presents high radon concentrations which during night periods could go up to 2000 Bq/m^3^. These concentrations represent a serious threat to the health of the occupants. Moreover, the other analyzed rooms present lower values that usually do not raise over 500 Bq/m^3^. Nonetheless, the radon concentrations also present values that go up more than the maximum allowed concentration within enclosed spaces [45]. Figure 12 is revealing that the highest radon concentrations were recorded for the period between 12:00 am–4:00 am when the occupants were asleep, and the windows closed–typical winter situation. Moreover, from the same measurements, it could be highlighted the moments when the windows had been opened and the fresh air coming from the outside replaced [46]. For instance, regarding the child’s bedroom, the window was usually opened around 09:00 am until 1:00 pm. During this time the radon concentration drops at around 150 Bq/m^3^ [47].

Figure 12 showed the evolution of the CO_2_ concentrations of the indoor environment during the experimental campaign. It could be revealed that the concentrations of CO_2_ are reaching higher values due to the presence of occupants and no fresh air [48]. Moreover, the parents’ bedroom reaches extreme CO_2_ concentrations because of the number of occupants. A similar pattern was observed for the CO_2_ concentration– huge accumulations during nighttime and rapid drop in the morning when the windows were opened.

**Figure 13:**
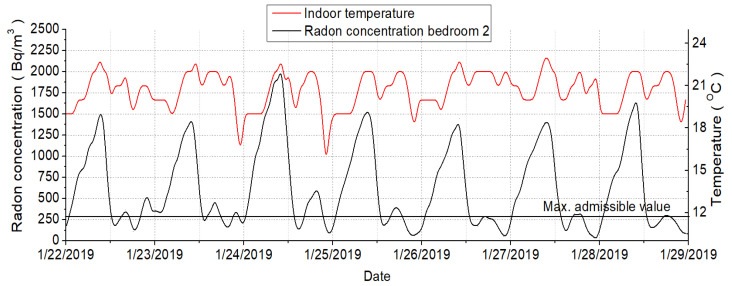
Correlation between radon concentration and air temperature.

Figure 13 highlights the correlation between the indoor air radon concentration and the interior air temperature regarding the bedroom occupied by the child. These results reveal that during night periods when the fresh air intakes are low, the radon concentration grows as the indoor or temperature is almost constant or with very small variations, whereas, during mornings when the windows are being opened, the radon concentration and the air temperature are dropping if the air change rates are higher.

As can be seen in the graphs above, the radon concentration is strongly connected or, the low radon values coincide with lower temperature values since both are influenced by ventilation. Otherwise, it seems that the higher temperature values coincide with the higher radon values, the temperature being responsible for that. When the dwelling is naturally ventilated, the indoor radon concentration, indoor temperature, and CO_2_ level decrease highly. In the accumulation period, when no natural ventilation is ensured, the temperature increase until reaches its setpoint, and the radon and CO_2_ concentrations increase highly until the natural ventilation is again ensured.

## 5. Research on the Optimal Solution

As was mentioned in the previous chapter, the decrease of the radon level inside the analyzed house can be achieved through natural ventilation, by opening the windows. This method is energy inefficient because all the available energy in the ventilated air is lost and not recovered at all. The solution was the implementation of a decentralized mechanical ventilation system combined with a minimally invasive system of active sub-slab-depressurization. The remediation system is based on the installation of simple-flow mechanical ventilation equipment, containing a heat recovery system, mounted in the outer wall. The operation of this equipment is continuous. For about 70 s it evacuates air from the house, while the heat is accumulated in the heat exchanger. After that, the fan reverses its rotation, introducing fresh air into the room, which takes the heat from the heat recovery. Details about the technical characteristics are found in Table 3.

The second remediation system is the installation of an active depressurization system of the basement with minimally invasive installation, by introducing the suction pipes from the outside without destroying the interior floor.

The pressure difference between the space under the floor and the interior is made by installing some manifold pipes, which are connected to an outlet pipe. An exhaust fan is installed at the end of this piping system. Details about the technical characteristics are found in Table 4.

**Figure 14:**
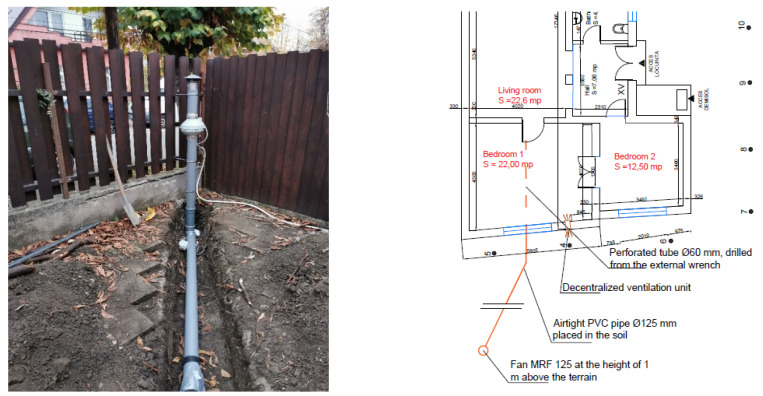
Layout and photo during the installation of the active depressurization system.

The main advantages of using the decentralized ventilation system are its compact dimensions, which offer the possibility to hide the entire equipment in the wall thickness. The high airflow is sufficient to assure good ventilation in the dwelling. Using the same route for both introduction and evacuation, a ceramic heat exchanger is suitable for use. The efficiency of heat recovery is up to 93%. The installation of this equipment can be made very easy by making a hole with a diameter corresponding to the model and fixing it (Figure 14). Due to its simple operating principle the equipment is energy efficient. The control of the system is done by remote control, manual regulator, or automatically by interconnection with the continuous measurement system. For minimal architectural impact on the building, the inlet/outlet vents will be protected with grilles. The main advantages of using the active depressurization system:

➢ Relatively small investment.
➢ Simple installation, from the outside, without damaging the interior.

**Figure 15:**
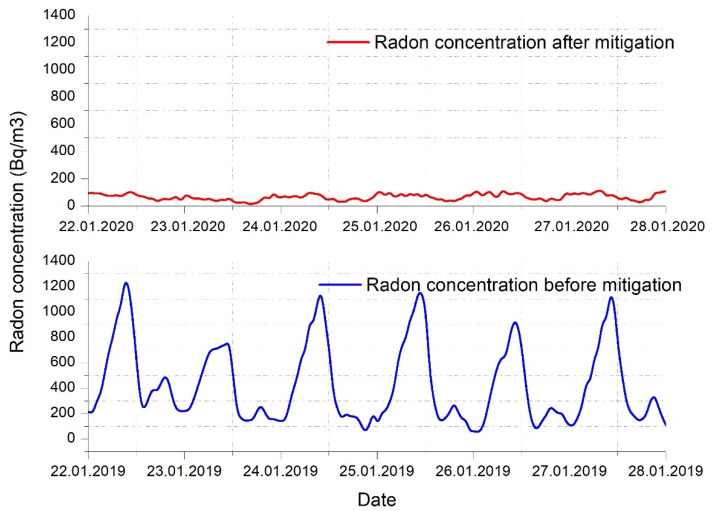
Correlation between radon level before and after mitigation applied.

**Figure 16:**
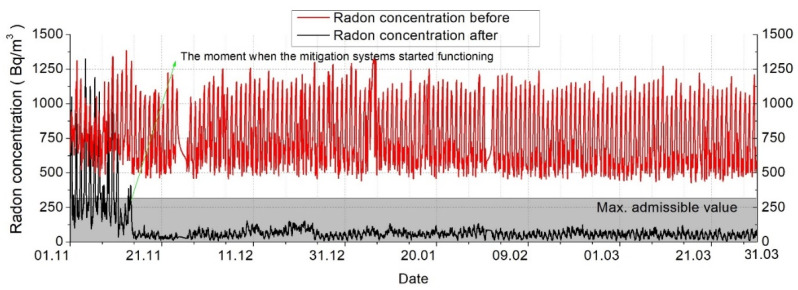
Correlation between radon level before and after the mitigation applied (long-range).

The fan can become noisy at high flow rates, but the system was installed by keeping a distance from the home. The second remediation system was installed because it was not possible to mitigate the radon level in the home using only one, so the operation of the two systems can be done simultaneously, according to the values recorded by the measuring device and control. The results of the two implemented remediation systems were represented and described in the following charts (Figure 15 and Figure 16), reflecting the need for their implementation.

As can be seen in Figure 15 and Figure 16, the radon concentration has been reduced drastically. Before any mitigation method was applied, extreme radon values were recorded, reaching frequently 1300–1400 Bq/m^3^. As can be seen, important fluctuations were recorded daily, due to the temperature differences and natural ventilation occurring. In the first days exposed in Figure 16, we can clearly see the commissioning of the mitigation systems, and how fast the radon concentration decreased to a normal value.

The implementation of this solution has as a preliminary result the reduction of radon concentrations in the analyzed dwelling below the normed limits, reaching a radon level reduction efficiency of approximately 86% (see Figure 16).

**Figure 17:**
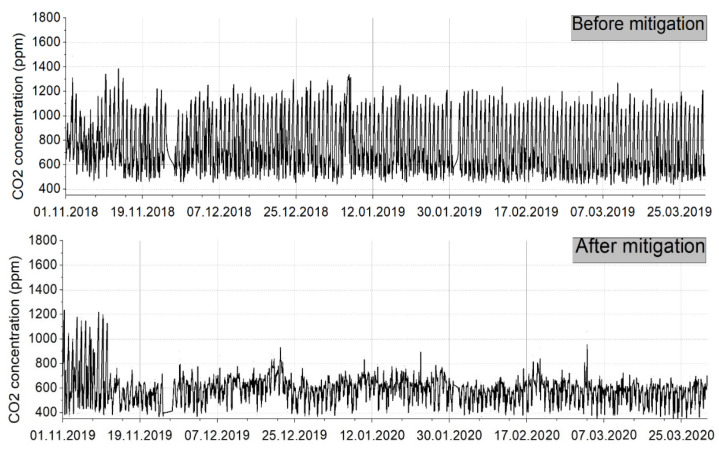
Carbon dioxide before and after the implementation of the HRV (long-range).

The graph below (see Figure 17) shows the current level of CO_2_ concentration in the analyzed house, compared to the measurements made during the same period last year when the systems for improving the air quality inside the analyzed house were not installed. The fluctuations of the CO_2_ concentrations in the analyzed house appear because every morning the rooms were ventilated by opening the windows. The maximum values were reached during the night when there was no fresh air source. After the measures to improve the indoor air quality have been applied, a mitigation of the level of CO_2_ concentration in the house can be observed, the values stabilizing at around 750 ppm. For a good evaluation of the efficiency of the implementation of this system in the analyzed dwelling, it is necessary to analyze the radon level for at least one year.

## 6. Mathematical and Numerical Determination

The indoor radon concentration could be determined also using the following formula:

(1) $$C=\frac{EA}{V\left(\lambda_{Rn}+\lambda_{v}\right)}$$

where ***C*** (Bq/m^3^) represents the indoor radon concentration, ***E*** (Bq/m^2^·h) is the exhalation rate, ***A*** (m^2^) is the radon exhalation surface, ***V*** (m^3^), represents the volume of the analyzed house, $\lambda_{Rn}$ (2.1 × 10^−6^ s^−1^) is the radon decay constant and $\lambda_{v}$ (h^−1^) represents the air change rate of the analyzed space.

During the experimental determination, the radon exhalation rate was established for the house ***E*** = 108 Bq/m^2^h. As the child’s bedroom presented the highest radon concentrations, from now on we only focused on this room. Using the formula above we wanted to determine the indoor radon concentration for different air change rates as follows: 0, 0.1, 0.2, 0.3, 0.4, 0.5 h^−1^ and their impact on the energy consumption. Hence, for a period of one typical winter week, it was studied the energy consumption for the mentioned air change rates from the dynamic point of view by means of simulations. The calculations considered an average indoor temperature of 21 °C, according to occupant’s preferences, and a mean outdoor temperature of −1.2 °C, with the lowest point of −14.8 °C, wet bulb temperature.

**Figure 18:**
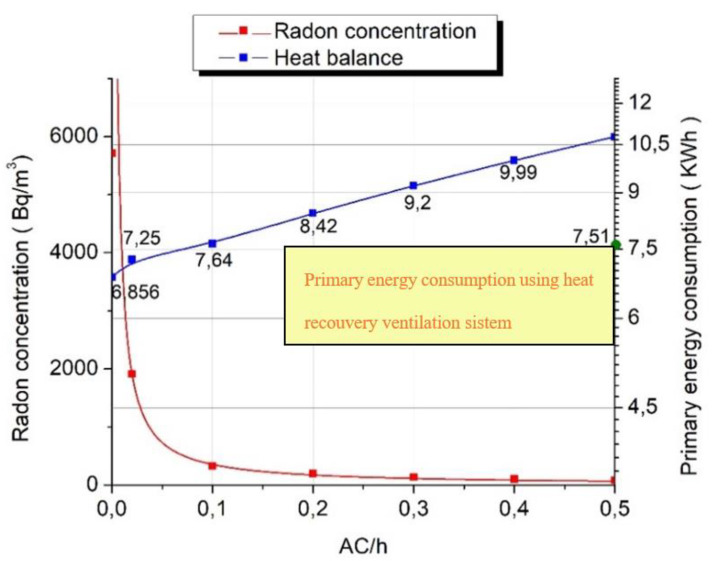
Indoor radon concentrations and primary energy consumption for one week.

Figure 18 presents the impact of the air changes per hour for both indoor radon concentration and primary energy consumption for our room determined for the same period as the measurements. It can be observed that a 0.1 h^−1^ could greatly impact the indoor radon concentration lowering its value around 350 Bq/m^3^ [49]. An 0.2 h^−1^ seems to lower the concentration enough to be under the maximum indoor limits, with a rise in the energy consumption of around 13%. From this moment, the growth of the air change rate does not have an important impact on the radon concentrations and it could be seen that a drop of around 50% in the radon concentration, corresponds to a 9% rise in energy consumption. It is important to understand that these primary energy consumptions were determined only for the five days of measurements and thus.

Table 5 reveals the primary energy consumption needed during the heating period of an entire year to maintain the indoor radon concentrations below the maximum indoor values. Therefore, an air change rate of 0.2 h^−1^ corresponds to the energy consumption of around 3,464,791 kWh, meaning a raise of the energy consumption of around 18% more than the normal case without any air changes. Moreover, obtaining an improvement in the indoor air quality through an air change rate of 0.5 h^−1^ results in a rise of around 35% of the heating load.

## 7. Conclusions

This paper aimed to evaluate the impact of the remedial system on the indoor air quality and energy consumption of a building. Most of the energy efficiency measures involve replacing the old wood-frame windows with double glazing PVC better in thermal performances but also better air sealing the house. The indoor air quality was not a serious problem 20 or 30 years ago when the buildings allowed a fresh air change through the doors or windows. The analyzed studied house is proof that a low-energy building without a controlled mechanical ventilation system can have serious problems in terms of indoor air quality.

(1) The novelty of this study came from the interconnection of radon mitigation methods with indoor air quality and building energy efficiency. Many studies have shown that radon mitigation by a Sub-slab depressurization system may reach efficiency values of about 95%. This method mitigates the radon concentration indoor, but, in some cases, this method cannot be applied. Another solution to mitigate the indoor radon level, when the concentration is not too much exceeded, may be the installation of ventilation equipment. This method showed a radon reduction efficiency between 25–67%.
(2) The simulations and the energy certification of the building demonstrated that the heating demand is low, and the building can be classified as an energy-efficient one. On the other hand, the indoor measurements conducted during the winter period have shown high radon concentrations up to 2000 Bq/m^3^ for a particular room during nighttime when the house was occupied, and air sealed as the windows were completely closed. After the mitigation method has been applied, the radon level decreased below 150 Bq/m^3^, showing a very good improvement for the indoor air quality.
(3) Using the mitigation systems, the temperature stabilizes at the setpoint, 21 °C, not oscillating anymore to low values. While the indoor air temperature presented a comfortable value and the heating system was functioning in good energy parameters, the indoor relative humidity established to a normal level of 750 ppm, high values like before the installation of mitigation system were not recorded anymore.
(4) Another great advantage of using this mitigation system is the reduction of energy loss, showing approximately 86% efficiency of the ventilation equipment (average known efficiency).

This paper proposes to highlight the fact that a balance between energy consumption and indoor air quality is needed, measuring the indoor air quality and eventually finding a remediation solution after a building is thermally retrofitted (walls, ceiling, windows).

## Figures and Tables

**Table 1:** Total exterior surfaces of the pilot building.

Surface	[m^2^]
Construction surface	137.98
Total area	275.95
Heated area	239.96
Heated volume (total)	599.90
Wall exterior surface	231.66
Total glazed exterior surface	31.76
Roof surface	137.98
Floor surface in contact with basement/ground	137.98

**Table 2:** Technical specifications of the TSR2 radon sensor.

Measurement sensitivity	0.15 count/hour/Bq·m^3^
Measuring range	5–65,535 Bq·m^3^
Measuring uncertainty	15% at 300 Bq per 1 h
Measuring relative humidity range	10–90%
Radon concentration results display	Short-term (1 h running average) Long-term (24 h running average)
Measurement sensitivity	0.15 count/hour/ Bq·m^3^

**Table 3:** Technical characteristics of the decentralized ventilation system.

Intake/exhaust airflow	20/40/60 m^3^/h
Acoustic pressure	10/18/26 dB(A)
Heat exchanger efficiency	Up to 93%
Control system	automatic
Voltage supply	230/50 Hz
Filter class	G4/F5/F7
Maximum energy consumption by fans	1.4, 2.3, 3.8 W

**Table 4:** Technical characteristics of the active sub-slab depressurization system.

Exhausted airflow	205 m^3^/h
Material	plastic
Intake/exhaust pipe diameter	125 mm
Control system	automatic
Voltage supply	230 V/50 Hz
Maximum fans consumed power	58 W

**Table 5:** Results regarding the primary energy consumption for the entire winter period in correlation with different air changes per hour.

Air Change Rate	The Primary Energy Consumption	
	Winter Period	Typical Winter Week
h^−1^	kWh	kWh
0	2,665,690	115,184
0.1	2,970,470	128,353
0.2	3,464,791	149,713
0.3	3,580,031	154,692
0.4	3,884,811	167,862
0.5	4,189,592	181,031

## Data Availability

The data used in this study can easily be accessed at the link: http://app.smartradon.ro, accessed on 24 November 2021.
